# Characterizing Individual Differences in Sweet Taste Hedonics: Test Methods, Locations, and Stimuli

**DOI:** 10.3390/nu14020370

**Published:** 2022-01-15

**Authors:** May M. Cheung, Matthew Kramer, Gary K. Beauchamp, Sari Puputti, Paul M. Wise

**Affiliations:** 1 Monell Chemical Senses Center, 3500 Market St., Philadelphia, PA 19104, USA; beauchamp@monell.org (G.K.B.); pwise@monell.org (P.M.W.); 2 Beltsville Agricultural Research Center, United States Department of Agriculture, 10300 Baltimore Ave., Beltsville, MD 20705, USA; matt.kramer@usda.gov; 3 Functional Foods Forum, Faculty of Medicine, University of Turku, 20014 Turku, Finland; sapupu@utu.fi

**Keywords:** sweet taste, hedonics, individual differences, methodology, sugar, remote testing, home-usage testing

## Abstract

Sweetness drives the consumption of added sugars, so understanding how to best measure sweet hedonics is important for developing strategies to lower sugar intake. However, methods to assess hedonic response to sweetness vary, making results across studies difficult to integrate. We compared methods to measure optimal sucrose concentration in 21 healthy adults (1) using paired-comparison preference tracking vs. ratings of liking, (2) with participants in the laboratory vs. at home, and (3) using aqueous solutions vs. vanilla milk. Tests were replicated on separate days to assess test-retest reliability. Test-retest reliability was similar between laboratory and home testing, but tended to be better for vanilla milk and preference tracking. Optimal sucrose concentration was virtually identical between laboratory and home, slightly lower when estimated via preference tracking, and about 50% lower in vanilla milk. However, optimal sucrose concentration correlated strongly between methods, locations, and stimuli. More than 50% of the variability in optimal sucrose concentration could be attributed to consistent differences among individuals, while much less variability was attributable to differences between methods. These results demonstrate convergent validity between methods, support testing at home, and suggest that aqueous solutions can be useful proxies for some commonly consumed beverages for measuring individual differences.

## 1. Introduction

The appeal (hedonic response) of sweetness is a key driver of consuming foods high in added sugars [1,2]. Overconsumption of added sugars contributes to increased risk of obesity and related chronic illnesses such as type 2 diabetes mellitus and cardiovascular disease [3,4,5]. A number of leading health agencies have recommended a reduction in intake of added sugars to improve public health and prevent chronic disease [6,7]. To develop successful treatments or policies to reduce intake of sugar, it is vital to understand individual differences in sweet taste and how they interact with diet to drive consumption. Yet, research on the relationship between sweet taste, diet and health has been equivocal [8,9,10,11,12,13,14,15,16,17,18,19,20,21,22,23]. Some researchers have found significant associations between perception of sweetness and intake of sugars [19], carbohydrates [9,10,20], energy from sweetened beverages [16,21], total energy intake [20], and body composition [15]. Other researchers have found no associations [12,13,14,18,22,23]. Differences in outcomes may be due in part to differences in methods and stimuli used to measure sweet perception, and several recent reviews have identified a need to standardize procedures [2,11,15,24,25,26].

Measures of sweet perception range from tests of sensitivity (e.g., minimum amount of sweetener detected or recognized as sweet) to supra-threshold intensity (sweetness rated on various scales) and hedonic response (liking, preference, and related constructs) [18,19,20]. Though associations with consumption of sugar have been observed for all these types of measures, association with hedonic response is the most consistent and most promising [2,11,19]. Within hedonics, preference (the tendency to choose one item over another) and liking (the pleasure derived from an item) are two common classes of measures. People presumably prefer foods they like more, though other factors may play a role (e.g., perceived health benefits or price) [2]. The current study focuses primarily on these two common classes of hedonic measure, though sweetness intensity was also measured.

Measures of preference typically involve comparisons among stimuli that differ in sweetness [27,28,29,30]. The Monell Forced-Choice, Paired-Comparison Preference Tracking test included in The NIH Toolbox for Assessment of Neurological and Behavioral Function is a widely used example [31]. For each trial, participants taste a pair of aqueous solutions of sucrose selected from five total concentrations (0.09 to 1.05 M), and they must choose the more preferred concentration. Concentrations of stimulus pairs vary over an experimental session as described in the methods (see Section 2.5.2). Henceforth, this method is called Preference Tracking. This Preference Tracking test was chosen as a representative measure of preference.

Measures of liking typically involve subjective ratings, though the sweet stimuli, concentration(s), liking scales, and methods to summarize data vary [18,19,20]. To facilitate direct comparisons with Preference Tracking, the current study includes ratings of liking for the same five concentrations of sucrose used in the Preference Tracking test. A visual analogue scale (VAS) was chosen for ratings of liking, since most VASs are reliable and easy for participants to use [24,26,32]. Henceforth, this method is called the Rating Method. If the two techniques measure the same underlying variable, as we hypothesized, the most liked (highest rated) concentration should correspond to the most preferred concentration measured using preference tracking. However, few studies have directly compared the two approaches using the same participants to determine whether there is convergent validity between the methods.

A variety of stimuli have been used to determine sweetness preference and liking. Aqueous solutions of sucrose are perhaps the most commonly used because they are easy to prepare and yield highly reproducible data [33]. However, aqueous solutions take sweetness out of its natural context in foods and beverages. More realistic model beverages have also been used to study sweet hedonics, e.g., vanilla milk [34] and lemonade [13,35], and do not always yield results identical to those from aqueous solutions [19,36]. Regardless, there have been few direct comparisons between more realistic beverages and aqueous solutions in the same participants using multiple procedures and test settings to determine how the various methodological factors interact.

The emergence of the COVID-19 pandemic in early 2020 greatly heightened interest in testing without person-to-person contact, with some encouraging results [37,38,39]. Conducting tasting sessions with participants at home could lower participant burden, use of fossil fuels, and risks associated with repeated visits to the laboratory (including risk of infectious illness). Testing at home or in the field has yielded useful data [37,38,39], but some researchers suggest that environmental factors can influence emotional and sensory responses to test samples [37,40,41,42,43]. Test location may also interact with test methods and sample type [44]. For example, a previous study comparing a central location test and a home use test found that home tests influence the hedonic response to some, but not all stimuli [44]. In addition, the Preference Tracking test requires more intensive experimenter-participant interactions compared to the Rating Method because the stimuli presented in each trial depend on the stimuli and responses earlier in the test session. Thus, Preference Tracking at home will require participants to play a more active role in selecting and handling stimuli, which could in turn affect results, e.g., increase errors in stimulus selection or make blinding less complete. However, to the best of our knowledge, laboratory and home testing environments have not previously been compared for the Preference Tracking test.

The current methods study was undertaken to inform the effort to compare and standardize methods. Three main manipulations were conducted: (1) Hedonic response measurement Methods (paired-comparison judgments of preference vs. rated liking); (2) test locations (in the laboratory vs. at home, supervised via video conferencing); (3) two stimulus matrices (simple aqueous solution of sucrose vs. sucrose in vanilla milk). All participants were tested under all conditions. This allowed us to determine if the various methodological factors interact, e.g., whether differences between home and laboratory testing are general or particular to sensory methods or stimuli.

## 2. Materials and Methods

### 2.1. Ethics Statement

Study procedures were reviewed by an institutional review board (IRB) at the University of Pennsylvania (protocol # 844423) and were determined to be exempt (category 6, flavor evaluation of wholesome foods/ingredients). The study was conducted in accordance with the guidelines of the Declaration of Helsinki, and participants provided written, informed consent prior to engaging in study procedures.

### 2.2. Participants

We recruited women and men between the ages of 21 and 65 years old from the Greater Philadelphia area between January and June 2021. Participants were recruited using flyers and from a pool of previous Monell participants who opted to be contacted again for future studies. We included generally healthy adults free of chronic diseases. We excluded individuals with current and chronic illnesses (e.g., heart diseases, diabetes, HIV/AIDS, kidney diseases), those with a history of food allergies or sensitivities, and those who took medication daily (except for birth control). Participants were screened via a phone interview for inclusion and exclusion criteria prior to enrollment. Informed consent was obtained from each participant prior to all data collection.

### 2.3. Design

All participants were tested under all conditions. After an initial laboratory session dedicated to instructions and practice as described below, participants completed four sensory testing sessions. Two replicate sessions were conducted in a sensory testing facility at the Monell Chemical Senses Center (lab), and two were conducted with participants at home, guided via video conferencing (home). Eleven participants had alternating test settings as follows: lab, home, lab, home. Ten participants had the alternating settings of home, lab, home, lab. Assignment to the two orders was counter-balanced. In each session, participants completed both sensory tasks, i.e., Preference Tracking (i.e., paired-comparisons) and ratings (i.e., ratings of liking and intensity of sweetness). The order of the sensory tasks (Preference Tracking and Rating Method) was counter-balanced across replicate sessions. Within each sensory task, participants tasted both stimuli (aqueous solutions and vanilla milk). The order of the stimuli within the sensory task was randomized within sessions.

Safety measures were developed in consultation with the Monell Human Subjects Committee. Participants were screened for symptoms and known or suspected exposure the night before and the day of each visit. One participant was tested at a time, with at least 30 min between participants for aerosols to clear via the building ventilation (with UV filtration) and to disinfect the testing room. Participants wore masks while not tasting. The experimenter wore a dental gown, N-94 or N-95 mask, face shield, gloves, and hair covering. Social distancing was practiced to the extent possible. Neither experimenters nor participants reported symptoms or positive COVID-19 tests during this study.

### 2.4. Stimulus Materials

Stimuli were prepared using food-grade sucrose (Fisher Chemical, crystalline/NF, catalog # S3-500) dissolved in either Millipore^TM^ (MilliporeSigma, Burlington, MA, USA) filtered, distilled, deionized water or in vanilla milk. Aqueous solutions were prepared at the five concentrations used in the Preference Tracking test [27]: 0.09, 0.18, 0.35, 0.70, and 1.05 M. Vanilla milk consisted of 2% fat milk (Giant™ brand, Giant Food Stores, LLC, Carlisle, PA, USA, SKU# 688267008634), and vanilla extract (1 mL per liter; Nature’s Promise Organic, SKU# 688267156502). Milk and vanilla were purchased from a local grocery store. Milk was stored under refrigeration and never used past use-by dates. Sucrose concentrations in vanilla milk were 0.03, 0.12, 0.23, 0.47, and 0.70 M. These concentrations were selected based on pilot work to approximately match the sweetness of aqueous solutions at each step. Briefly, experimenters first tasted and adjusted concentrations of sucrose in vanilla milk to approximately match the sweetness of each of the five concentrations in the aqueous solutions. A group of 20 healthy adults with minimal training (just general Labeled Magnitude Scale(gLMS) instructions) rated the sweetness intensity of each concentration twice in blocked random order using procedures similar to those described in Section 2.5.3. No significant differences in sweetness intensity were found. Solutions were prepared using sterile glassware. Samples were presented as 10 mL aliquots in 30 mL plastic medicine cups and served cold, at approximately 4 °C.

For lab tests, experimenters poured samples out of participants’ sight. For home tests, participants poured 10 mL samples themselves, using graduated plastic medicine cups provided. Home test kits included twenty 120 mL bottles (two instances of each of the five concentrations of each stimulus, sufficient for one test session). Bottles were labeled with random alpha-numeric codes and packed in cooler bags with ice packs for transport. Lab and home sessions alternated, as described in Section 2.3, so participants received a test kit to take home at the end of lab sessions. Participants were instructed to take the kits directly home and refrigerate samples until the scheduled sessions. Samples were used within 5 days.

### 2.5. Procedures

#### 2.5.1. Training

The training session was dedicated to measurement of participant height/weight, collection of demographic information, and task instructions. Participants received standard instructions on the use of the gLMS for measuring sweetness intensity, then practiced by rating the intensities of real and imagined sensations [45]. The gLMS is a vertical scale with intensity descriptors as follows: “barely detectable,” “weak,” “moderate”, “strong”, “very strong” and “strongest imaginable sensation of any kind”, with the spacing of the descriptors on the scale determined empirically to be proportional to strength of sensation [42]. Next, participants were instructed in the use of a 100-point visual analogue scale (VAS) for rated liking (horizontal scale, anchored with “dislike extremely” on the left and “like extremely” on the right) [31]. Participants practiced by rating liking for remembered or imagined sensations.

#### 2.5.2. Preference Tracking

Procedures for the Monell Forced-Choice, Paired-Comparison Tracking Procedure have been published elsewhere [27]. Participants completed the Preference Tracking procedure for each stimulus during each session, with breaks of 5 min between Preference Tracking runs for the two stimuli. Participants began each session by rinsing the mouth four times with distilled water (Good & Gather^TM^, Target Brands, Inc, Minneapolis, MN, USA, SKU#: 085239047675). For each trial, participants tasted pairs of 10 mL samples of liquid presented in 30 mL plastic medicine cups, with a 1 min break between pairs. Participants then chose which stimulus they preferred. The first pair of samples were the from the middle of the range (0.18 M vs. 0.70 M for the aqueous solutions). Each subsequent pair contained the participant’s preceding preferred concentration paired with an adjacent stimulus concentration. This pattern continued until the participant chose two consecutive times either the same concentration of sucrose paired with both a higher and lower concentration or the highest (1.05 M) or lowest (0.09 M) concentration. The entire task was repeated after a 5 min break, with the stimulus pairs presented in reverse order. The procedure was repeated twice within a session, and the geometric mean of the two trials was defined as the most preferred concentration.

#### 2.5.3. Ratings of Sweetness and Liking

Participants sampled by taking the entire contents of the cup into the mouth, moving the liquid around in the mouth for several seconds, then rating liking and sweetness intensity in that order [24,45]. Stimulus presentation and tasting followed the procedures in Section 2.5.2. Next, participants expectorated the sample and rinsed with water at least twice to begin a 1 min pause before the next sample. Each sensory test session included 20 trials, separated into two blocks of 10 (all five concentrations for a given stimulus in random order, then again in random order). A 5 min break separated blocks for the two stimuli.

### 2.6. Data Analysis

#### 2.6.1. Sensory Endpoints

The measures of main interest included optimal sucrose concentration estimated via Preference Tracking (most preferred) and optimal sucrose concentration estimated via the Rating Method (most liked) at different locations (lab vs. home) in two stimulus matrices (aqueous solution vs. vanilla milk). Methods for calculating most preferred concentration have been described previously [27]. Most liked was defined as the concentration associated with the maximum (among presented concentrations) in rated liking. To find maxima, rated liking was plotted against the cube root of sucrose concentration (to space concentrations approximately equal distances apart). Functions of liking vs. transformed concentration were fitted using stepwise regression (lowest BIC criterion), up to a cubic polynomial. If the resulting model was intercept only (flat), the most liked concentration was defined as the geometric mean of the two concentrations associated with the highest ratings (see Supplementary Material Table S1 for fit parameters). These parametric estimates of most liked concentration correlated strongly (r = 0.86 for aqueous solutions, r = 0.94 for vanilla milk) with the concentration associated with the highest rating of liking; because the two methods for estimating most liked concentration supported the same conclusions, only values from model fits are reported.

Most preferred and most liked concentrations were both positively skewed. The best common Box-Cox power transformation (λ = 0.22) was used for both Preference Tracking and the Rating Method prior to inferential analysis. Mean values of optimal sucrose concentration, defined as the most preferred or the most liked concentrations from Preference Tracking or the Rating Method, were back-transformed and reported in the original units (molar concentration of sucrose). Rated sweetness intensity (gLMS) was of secondary interest. Ratings made using the gLMS were also positively skewed. The optimal Box-Cox power transform (λ = 0.47) was used for inferential analysis for sweet intensity. Mean values were back-transformed and reported in units ranging from 0 (“no sensation”) to 100 (“strongest imaginable sensation of any kind”).

#### 2.6.2. Statistics

Test-retest reliability between replicate sessions was evaluated using Pearson’s correlation coefficients (r). To examine differences in test-retest reliability between locations (lab vs. home), stimuli (aqueous solution vs. vanilla milk), and methods (Preference Tracking vs. the Rating Method) for estimating optimal sucrose concentration, we tested pairs of partial correlation coefficients using the Fisher r-to-z transformation. For example, to assess whether optimal concentration is more reliable in the laboratory than at home, the partial correlation (across participants, adjusting for stimulus and method) between session 1 and session 2 in the laboratory was compared to the corresponding partial correlation between session 1 and session 2 at home. Partial correlations were tested in the same way for method and stimulus. Repeated measures analysis of variance (ANOVA) was used to assess the effects of experimental conditions on average values of sensory measures. ANOVA models included main effects and all second-order interactions. Overall variance in optimal concentration was decomposed using a linear mixed model with all effects random (to estimate variances). The main effects were method, stimulus, location, and participant. All second-order interactions were also included. The residual terms contained within subjects variability, unexplained variability (not due to included terms, such as higher order interactions or unmeasured independent variables), and model error. Analyses were conducted using the lme4 package [46] in R (version 3.6.2., R Development Core Team, 2021) and Microsoft Excel (Version 16.52, Redmond, WA, Microsoft Corporation). All results, unless otherwise indicated, were reported in mean ± standard deviation or (95% confidence interval).

## 3. Results

### 3.1. Participant Characteristics

Twenty-one healthy adults between the ages of 21 and 49 (33.1 ± 10.0 years) participated. Eleven self-identified as female, and the rest self-identified as male. Self-identified racial demographics were as follows: 16 Caucasian; 3 African-American/Black; 1 Asian; 1 multi-racial. Average body mass index (BMI) was 29.6 ± 7.8 kg/m^2^. Four participants were normal weight (BMI < 25 kg/m^2^); ten were overweight (BMI between 25 to 30 kg/m^2^), and seven were obese (BMI ≥ 30 kg/m^2^).

### 3.2. Hedonic Measures

#### 3.2.1. Test-Retest Reliability

Test-retest reliabilities were assessed to determine the stability of hedonic response measures across replicate sessions within subjects. For rated liking, averaged across concentrations, test-retest reliability coefficients were r = 0.66 ± 0.13 and 0.64 ± 0.11 for aqueous solutions tested in lab and at home, respectively. Average test-retest reliability coefficients were r = 0.67 ± 0.16 and 0.72 ± 0.11 for vanilla milk tested in lab and at home, respectively. Test-retest reliability for optimal concentrations derived from Preference Tracking (most preferred) and ratings of liking (most liked, as described in Section 2.5.3) were of comparable strength (Table 1), though test-retest reliability was lower for the most liked concentration in aqueous solutions.

Differences in test-retest reliability between test locations were not statistically significant (*p* = 0.72). Reliability for vanilla milk was significantly greater than for the aqueous solutions (*p* = 0.02). Preference Tracking tended to be more reliable than the Rating Method, though this difference was statistically marginal (*p* = 0.06).

#### 3.2.2. Mean Values of Optimal Concentration

**Figure 1:**
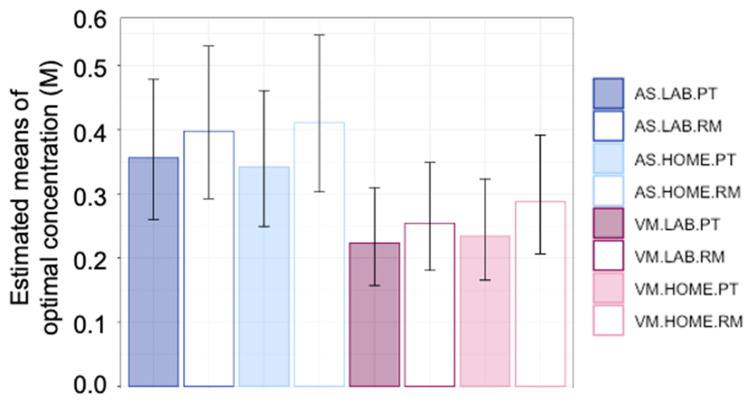
Optimal sucrose concentrations, averaged across replicate test sessions. Values are means estimated from a repeated-measures ANOVA on Box-Cox transformed sucrose concentrations, then back-transformed to the original units (M). AS = estimated using aqueous solutions; VM = estimated using vanilla milk; lab = test conducted in the laboratory; home = test conducted with participants tasting at home, supervised via video conferencing; PT = Preference Tracking, or optimal (most preferred) concentration estimated via paired-comparison preference tracking; RM = Rating Method, or optimal (most liked) concentration estimated from ratings of liking. For example, AS.LAB.PT is optimal concentration estimated using aqueous solutions, in the laboratory, via paired-comparison preference tracking. Error bars represent 95% confidence intervals.

Individual participants displayed different patterns of rated liking over concentrations (Supplementary Materials Figures S1 and S2). Descriptive statistics for the untransformed data are represented in Supplementary Material Figure S3. Differences in optimal sucrose concentrations between experimental conditions for individual participants are depicted in Figure S4. A four-way ANOVA was performed on Box-Cox transformed optimal concentration: method (Preference Tracking vs. the Rating Method) X location X stimulus X session, with Participant as a random effect. Optimal concentration estimated by the Rating Method was slightly but significantly higher than Preference Tracking (Figure 1); F(1, 305) = 10.03, *p* = 0.002. There was a significant main effect of stimulus; F(1, 305) = 69.35, *p* < 0.001. Optimal concentration tended to be lower in vanilla milk than in the aqueous solutions (Figure 1). Finally, there was a significant stimulus X session interaction; F(1, 305) = 4.90, *p* = 0.028. Averaged across other conditions, optimal concentrations in the aqueous solutions differed little between the first (0.39 M; 95% CI: 0.29, 0.51) and second sessions (0.37 M; 95% CI: 0.26, 0.51). However, for vanilla milk, optimal concentration increased slightly from the first session (0.23 M; 95% CI: 0.16, 0.31) to the second session (0.27 M; 95% CI: 0.19, 0.37). No other effects reached significance (0.10 < *p* < 0.94). Thus, mean optimal concentration did not differ between location (lab and home), nor was testing location or its interactions with other variables significant. Optimal concentration differed by method and stimulus, but the only interaction was a tendency for optimal concentration to increase slightly from session 1 to session 2 and only for vanilla milk.

#### 3.2.3. Correlations between Conditions across Individuals

**Figure 2:**
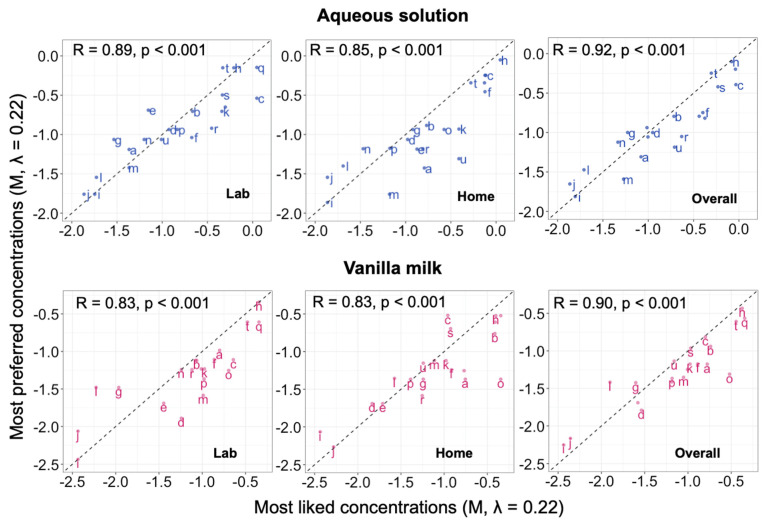
Scatter plots for optimal sucrose concentration estimated via paired-comparison preference tracking (most preferred, averaged across replicate sessions) vs. optimal concentration estimated from ratings of liking (most liked). Values are Box-Cox transformed (λ = 0.22) sucrose concentrations (M). The letters on the plot represent participant ID. Top (blue points): data for aqueous solutions. Bottom (pink points): vanilla milk. Left to right: measured in the laboratory, measured at home, and averaged across the two test locations (overall). Dotted line: line of equality (y = x line).

**Figure 3:**
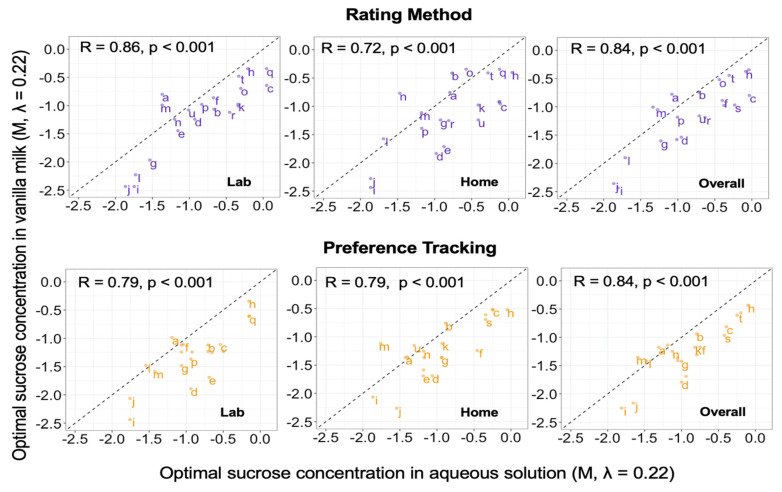
Scatter plots for optimal concentration measured using vanilla milk (averaged across replicate sessions) vs. using aqueous solutions. Values are Box-Cox transformed (λ = 0.22) sucrose concentrations (M). The letters on the plot represent participant ID. Top (purple points): optimal concentration estimated from ratings of liking (Rating Method). Bottom (orange points): optimal concentration estimated via paired-comparison preference tracking. Left to right: optimal concentration measured in the laboratory, at home, and averaged across the two test locations (overall). Dotted line: line of equality (y = x line).

**Figure 4:**
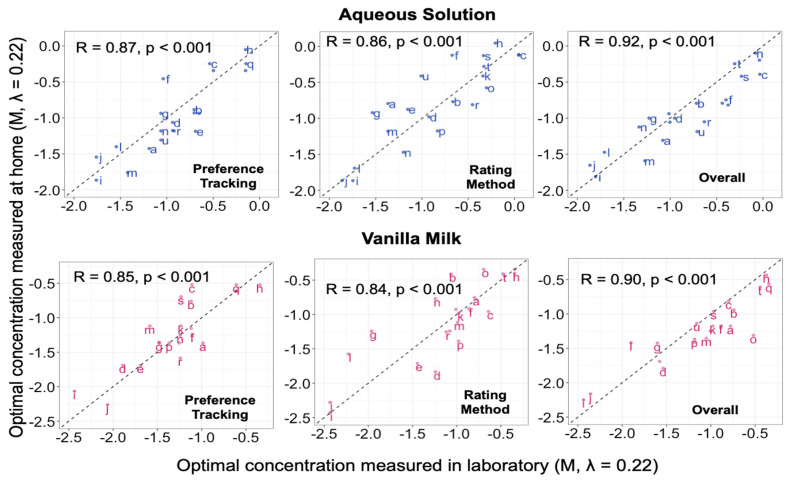
Scatter plots for optimal concentration measured at home (averaged across repeated sessions) vs. optimal concentration measured in the laboratory. Values are Box-Cox transformed (λ = 0.22) sucrose concentrations (M). The letters on the plot represent participant ID. Top (blue points): optimal concentration estimated using aqueous solutions. Bottom (pink points): optimal concentration estimated using vanilla milk. Left to right: estimated via paired-comparison preference tracking (PT), estimated from ratings of liking (RM), and averaged across the two sensory methods (overall). Dotted line: line of equality (y = x line).

Correlations between optimal concentrations measured under the various experimental conditions (using Preference Tracking vs. the Rating Method, in the lab vs. home, and using aqueous solution vs. vanilla milk) were positive. The average correlation between optimal concentration measured with at least one difference in conditions (r = 0.68 ± 0.11) was comparable to the average test-retest reliability (r = 0.71 ± 0.13, partial correlations 0.66 (95% CI: 0.56, 0.74)). Averaging across replicate sessions, correlations between most preferred and most liked concentrations ranged from 0.83 to 0.89 (partial correlation r = 0.76 (95% CI: 0.68, 0.82)) (Figure 2). Correlations between lab and home ranged from 0.84 to 0.87 (partial correlation r = 0.75 (95% CI: 0.67, 0.81)) (Figure 3). This suggested that tests conducted at the two locations provided overlapping information on individuals. Finally, correlations between optimal concentrations measured using aqueous solution and vanilla milk ranged from 0.72 to 0.85 (partial correlation r = 0.68 (95% CI: 0.58, 0.75)) (Figure 4). This suggested that both model stimuli provided overlapping information on individual differences in hedonic judgments.

In the variance decomposition analysis, the participant X method, participant X stimulus, and participant X session two-way interactions accounted for between 3 and 6% of variance, suggesting that individuals differed slightly in their response to testing methods (Table 2). However, the individual-to-individual variability by itself (Participant) accounted for by far the largest proportion of total variance, about 51%. Accordingly, though optimal concentration differed between methods and stimuli (see Section 3.2.2), consistent differences among individuals had a larger effect on optimal sucrose concentration than the experimental main effects.

### 3.3. Rated Sweetness Intensity

#### 3.3.1. Test-Retest Reliability

Test-retest reliabilities were assessed to determine the stability of intensity ratings across replicate sessions within subjects. For the aqueous solutions, average (across concentrations) test-retest reliability coefficients were similar between test locations (lab r = 0.65 ± 0.08; home r = 0.64 ± 0.14). For vanilla milk, average test-retest reliability was also comparable between locations (lab r = 0.50 ± 0.30; home r = 0.56 ± 0.16). A four-way, repeated measures ANOVA was performed on transformed ratings: location X stimulus X Sweetness Level (the five concentrations per stimulus) X session, with Participant as a random effect. There was a significant main effect of session; F(1, 1637) = 11.53, *p* < 0.001. Ratings were lower overall in session 1 (19.29; 95% CI = 16.35, 22.36) than in session 2 (19.76; 95% CI 16.94, 22.83). A significant location X session interaction; F(1, 1637) = 6.27, *p* < 0.02, reflected a difference between sessions in the laboratory: Ratings were slightly lower in session 1 (18.75; 95% CI = 15.53, 22.29) than in session 2 (20.13; 95% CI 17.11, 23.42). There were no differences between session 1 (19.53; 95% CI = 16.24, 23.14) and session 2 (19.54; 95% CI 16.56, 22.76) at home. Thus, differences between sessions were small in practical terms and suggest greater stability at home. No other effects involving location reached significance. In general, there was good agreement between lab and home tests (agreement between locations also held at the individual participant level; see Supplementary Materials Figures S5 and S6).

**Figure 5:**
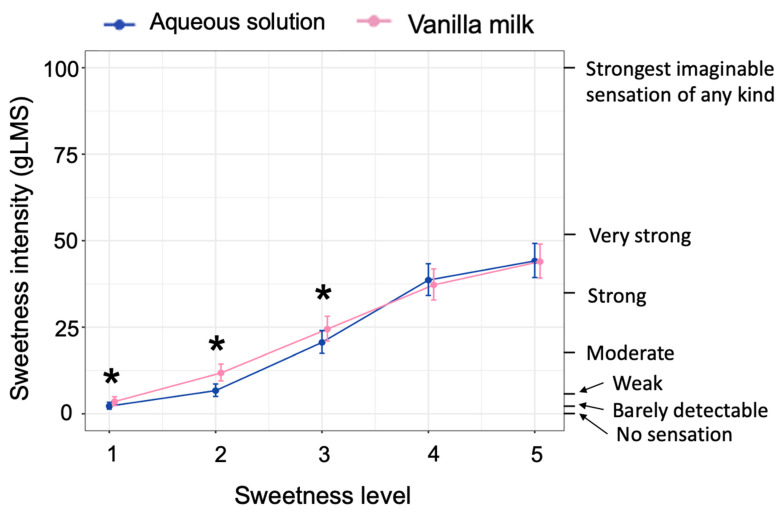
Average back-transformed ratings of sweetness intensity made using the general Labeled Magnitude Scale (gLMS). Data are separated by stimulus, averaged across other conditions (blue symbols for aqueous solutions, pink symbols for vanilla milk). * indicates a significant difference in rated intensity between stimuli at a particular concentration-step (*p* < 0.05, according to Bonferroni-corrected post hoc contrasts). Note that back-transformed values at the lowest concentration differ very little, but the Box-Cox transformed values (λ = 0.47) on which analyses were conducted magnify differences for very low values. Error bars represent 95% confidence intervals.

The effect of Sweetness Level was significant; F(1, 1637) = 1422.86, *p* < 0.001, an expected dose-response relationship (Figure 5). The effect of stimulus was also significant; F(1, 1637) = 39.42, *p* < 0.001. Ratings were slightly lower overall for aqueous solutions (18.14; 95% CI = 14.99, 21.62) than for vanilla milk (20.90; 95% CI 17.81, 24.25). Furthermore, there was a significant stimulus X Sweetness Level interaction; F(1, 1637) = 12.37, *p* < 0.001. Rated intensity covered a similar range for the two stimuli, but vanilla milk was sweeter for lower concentration steps (Figure 5). Other effects were not significant. Thus, sweetness covered a similar range for aqueous solutions and vanilla milk, and sweetness was approximately, though not perfectly, matched at individual concentration steps.

#### 3.3.2. Correlations between Sweetness Intensity and Optimal Concentration

To determine if individual differences in sweetness intensity were associated with individuals’ optimal concentration, average (across concentrations) sweet intensity was calculated as a measure of sweet taste sensitivity. The rated intensity for aqueous solutions was not significantly (*p* > 0.05) correlated with either the most preferred (r = 0.07) or most liked (r = 0.07) concentration in aqueous solutions. Similarly, the rated intensity for vanilla milk was not significantly correlated with either the most preferred (r = −0.10) or most liked (r = −0.09) concentration. Accordingly, the data analyses provide no evidence that individual differences in rated sweetness intensity are associated with individual estimates of optimal concentration of sucrose.

## 4. Discussion

### 4.1. Summary of Major Findings

The current study assessed the effects of method (Preference Tracking vs. Rating Method), test location (lab vs. home), and stimulus (aqueous solution vs. vanilla milk) on optimal concentration of sucrose in model beverages. Between methods, test-retest reliability for most preferred (Preference Tracking) and for most liked (Rating Method) concentration did not differ significantly, although the former tended to be greater than the latter. Optimal concentration across participants tended to be slightly higher for the Rating method than for Preference Tracking, but the two measures were strongly correlated across individuals. In terms of location, results were essentially identical between test locations in terms of test-retest reliability, mean values, and patterns of individual differences. For stimulus, the optimal sucrose concentration averaged across participants was higher in aqueous solutions than in vanilla milk (by about 50%, on average), but values for the two stimuli were strongly correlated across individuals. Test-retest reliability was significantly greater for vanilla milk than for aqueous solutions. Rated sweetness intensity was similar between lab and home for both stimuli. Overall, the results suggested that all methods tested in the current study captured the same latent variable in optimal sucrose concentration in model beverages.

### 4.2. Preference Tracking vs. the Rating Method

Judgments of preference and ratings of liking are different tasks for measuring sweet hedonics, but they captured similar person-to-person differences. Results from the two methods (Preference Tracking vs. the Rating Method) correlated strongly (0.83 ≤ r ≤ 0.89), and the variance decomposition suggested that method accounted for only about 1% of total variance in selecting the optimal concentration, compared to consistent differences among individuals, which accounted for about 51% of the total variance, i.e., method was not a significant contributor to the differences in optimal concentration. These findings confirm those of Asao and colleagues, who found a strong association between the most preferred and most liked concentration of sucrose in aqueous solutions using similar methods [47]. The current study extends those findings to show associations of comparable strength across two stimuli and testing environments. Other studies, which did not focus on optimal concentration for both tasks, found that the most preferred concentration differed across sweet-liker phenotypes, defined according to the overall shapes of functions of rated liking vs. concentration [28]. Another study found that rejection thresholds, or the concentration differences at which people choose a low sugar formulation of orange juice over a higher sugar formulation in preference judgements, were only measurable for people whose ratings of liking decreased at high concentrations [48]. Our results add to the accumulating evidence that judgments of preference and of ratings of liking are related. Here, tests used the same stimulus, with the same participants, tested at the same time. Agreement between Preference Tracking and the Rating Method might be weaker if the tests also differed in stimuli, concentration range, number of concentration steps, or other factors. However, these finding suggest that studies of individual differences in sweet hedonics that use paired-comparison preference judgements and ratings of liking could be meaningfully integrated in reviews or meta-analyses.

Ratings of liking may yield higher optimal concentration compared to judgements of preference. Note that the difference between the methods was small and consistent across test locations and stimuli, which suggests that an adjustment factor could be applied if one desired to use one test as a proxy for the other. Preference Tracking was slightly more reliable and may provide practical advantages in some situations. For both methods, test-retest reliability in the current study fell well within the range previously reported using similar methods [20,27,47,49,50]. The small difference in test-retest reliability between the methods may mean more replicate measures using the Rating Method might be required to match the test-retest reliability of Preference Tracking.

### 4.3. In-Laboratory vs. At-Home Tests

Consistent with other studies, our findings show that sensory tests conducted outside a carefully controlled laboratory setting are feasible and generally comparable to laboratory tests [37,38,39,44,51,52,53,54]. For example, a study by Seo and colleagues compared data from drive-in sensory booths to data from the laboratory in 106 consumers [38]. Hedonic and emotional responses to model beverages were not statistically different between testing conditions [38]. Another study conducted by the Italian Sensory Science Society compared laboratory and remote (at work and at home) sensory tests, including ratings of liking with multiple food items, and found similar results between test locations [37]. With adequate guidance by experimenters via video conferencing, most remote tests (including ratings of liking) yielded data comparable to those from laboratory tests [37]. Consistent with these findings, test-retest reliability, mean values, and patterns of individual differences in optimal sucrose concentration were essentially identical between test locations in the current study, as was rated sweetness. There were no strong interactions between location and other test conditions, with the caveat that only second-order interactions were tested. Overall, the results suggested that, to a first approximation, hedonic response remained stable across test methods and stimuli, regardless of test location. Furthermore, variance decomposition suggested that test location and second-order interactions involving location accounted for little or no variance in optimal concentration. The current study extends previous findings to paired-comparison Preference Tracking of sweetness in two model stimuli. Thus, results suggest that remote evaluation of hedonic response to sweetness is feasible and comparable to laboratory tests. Limiting or eliminating visits to the laboratory could reduce participant burden, use of fossil fuels, and risk of exposure to infectious illnesses such as COVID-19. Remote testing would also expand the pool of potential participants beyond those available locally, which could in turn ease recruitment efforts and facilitate studies with more generally representative samples.

### 4.4. Aqueous Solutions vs. Vanilla Milk

The sample matrix used in sensory tests can affect hedonic responses [19,28,36,55,56]. The most commonly used stimuli for measuring individual differences in hedonic response to sweetness are simple aqueous solutions of sucrose [26,57]. However, aqueous solutions take sweetness out of the natural context of foods and beverages, which might affect patterns of individual differences. For example, Bertino and colleagues found that Taiwan-born students studying in the US tended to show higher preference for sucrose solutions than students of European descent, but preferred lower concentrations in cookies [36]. Similarly, Holt and colleagues found differences in rated liking for orange juice and biscuits between Malaysian-born and Australian-born students, but hedonic response to sucrose solutions did not predict these differences [19]. Thus, experience and culture may play a role in determining optimal sweetness in particular foods [33]. Furthermore, individual differences in sensitivity to sourness, bitterness, and astringency (sensations that are often ameliorated by sweetness) might interact with liking for sweetness to drive preference in foods and beverages in which these generally negative sensations are prominent [55,58]. However, the current study found that the optimal concentrations of sucrose were correlated between aqueous solutions and vanilla milk (0.77 ≤ r ≤ 0.85). The variance decomposition suggested that stimulus accounted for about 10% of total variance in optimal concentration (consistent with a significant mean difference between stimuli), and second-order interactions involving stimulus accounted for up to only 6%. Thus, within the sample of participants living in the Philadelphia area of the United States, aqueous solutions and vanilla milk provided comparable information on individual estimates of optimal sucrose concentration. This result suggests that individual estimates of optimal sucrose concentration in aqueous solutions can be generalized to some more realistic beverages [28], though based on past work, we expect that the results may not generalize to all model foods [59].

Although the hedonic results from the aqueous solutions and vanilla milk are strongly correlated, the overall optimal concentration of sucrose was substantially lower in vanilla milk than in the aqueous solution, an effect that was consistent across sensory methods and test locations. Previous studies have suggested that sweetness might interact with other flavor components of foods and beverages, including fat and aroma, to shape hedonic response [59,60,61]. In particular, vanilla flavor can enhance sweetness in both simple model solutions and dairy beverages in cultures and ethnic groups where vanilla is associated with sweet taste [34,62,63]. In addition, the slightly higher test-retest reliability in vanilla milk compared to aqueous solutions measured at home might be because it is easier for participants to choose an optimal concentration in a more familiar beverage context. Consistent with sweetness enhancement by vanilla, participants rated vanilla milk as equally sweet or even sweeter than aqueous solution at each concentration step, despite lower sucrose concentrations. It would be interesting to repeat the comparison between stimuli using nose-clips to block retro-nasal aroma to see if the optimal concentrations still differ between stimuli. The results might be relevant to the hypothesis that aroma can be used to partially compensate for reduced sugar levels in beverages [64].

### 4.5. Rated Sweetness Intensity

Rated sweetness was not associated with individual differences in optimal sucrose concentration. This result is consistent with many studies on sweet hedonics, which have found that sweet liking is weakly associated with perceived intensity of sweetness [16,24,28,65,66]. Despite good matching at each sweetness concentration step in a pilot study, the current sample of participants rated the lower concentration steps of vanilla milk as sweeter than the corresponding steps of aqueous solutions, though differences in rated sweetness were modest in magnitude. Though perceived sweetness covered a similar range for the two stimuli, the imbalance at lower steps is consistent with the lower optimal concentrations found in vanilla milk, as discussed above (see Section 4.4). However, the imbalances in intensity did not affect the correlation between individual optimal concentration between the two stimuli.

### 4.6. Limitations

This study has several limitations. First, this study has a modest sample size relative to many past studies of hedonic response to sweetness. The sample size was sufficient to discern modest differences in optimal sucrose concentration between test conditions and associations between methods in individual response. However, conclusions regarding apparent differences in correlations between methods and stimuli require confirmation in a larger sample. Another limitation is that the study included a limited array of methods. Only one realistic model beverage was compared to aqueous solutions. More beverages and foods, particularly solid foods, would be useful to evaluate the generality of the findings, but it would be difficult to include enough model foods in a single study to reach very broad conclusions. In addition, other endpoints related to hedonic response, e.g., wanting and purchase intent, could be incorporated in future studies. Furthermore, we only used five sucrose concentrations, considerably fewer than in some studies [16,24,47]. This might limit resolution of individual hedonic response, which could, in principle, obscure some differences between methods. Although the range of concentrations used was comparable to some other studies [13,19,24,28,47], different results might be obtained if higher concentrations of sucrose were used. Regarding the conclusion that home tests provide data comparable to laboratory tests, it should be noted that participants attended one in-person training session prior to their home testing sessions. Further work would be required to determine if remote training is equally effective. It would also be interesting compare results between trained vs. untrained participants. For large-scale implementation in population studies, even the single, brief training session used in the current study might be impractical, so it would be valuable to determine the minimal training required to obtain reliable results. Finally, test-retest reliability was assessed over a relatively short period of time, so conclusions may not generalize to longer periods.

## 5. Conclusions

The optimal concentration of sucrose in model beverages was virtually identical between in-laboratory and at-home tests and varied only slightly by method and stimulus. Individuals’ estimates of optimal concentration correlated strongly among the various conditions, suggesting that all methodological conditions ultimately captured a common underlying trait—sweet hedonic response. Furthermore, aqueous solutions can be useful proxies for some commonly consumed beverages for measuring individual differences in sweet hedonic response. These results suggest that reviews and meta-analyses on individual differences in optimal sucrose concentration can be conducted with studies using different methods. The choice of which method to use may depend on other factors, including target population. Regarding remote testing, we show that Preference Tracking via video conferencing is feasible and reliable. However, Preference Tracking requires focused, one-on-one interaction between an experimenter and participants, because the stimuli presented in each trial depend on previous stimuli and responses. Therefore, some variant of the rating method, which can use pre-ordered sets of stimuli, may be more practical and efficient for remote testing.

## Figures and Tables

**Table 1:** Test-retest reliabilities over two sessions (Pearson’s r) for ratings of liking and hedonically optimal sucrose concentration.

Aqueous Solution	Lab	Home
0.09 M	0.82	0.79
0.18 M	0.76	0.57
0.35 M	0.52	0.58
0.70 M	0.54	0.54
1.05 M	0.64	0.71
Pref ^a^	0.71	0.73
Liked ^b^	0.60	0.42
Vanilla Milk	Lab	Home
0.03 M	0.88	0.75
0.12 M	0.47	0.54
0.23 M	0.64	0.70
0.47 M	0.79	0.79
0.70 M	0.65	0.81
Pref ^a^	0.79	0.86
Liked ^b^	0.72	0.79

^a^ Most preferred, preference tracking; ^b^ Most liked, rating method.

**Table 2:** Results of variance decomposition of hedonically optimal concentration.

Source	Percent of Variance
Location	0.00
Method	1.24
Stimulus	11.11
Session	0.00
Participant	51.00
Location X Method	0.00
Location X Stimulus	0.00
Location X Session	0.21
Location X Participant	0.42
Method X Stimulus	0.00
Method X Session	0.46
Method X Participant	2.82
Stimulus X Session	0.91
Stimulus X Participant	6.10
Session X Participant	3.73
Residual	22.00

## Data Availability

De-identified data are available upon request from the corresponding author.

## Supplementary Materials

The following are available online at https://www.mdpi.com/article/10.3390/nu14020370/s1, Figure S1: Liking vs. concentration functions for individual participants, aqueous solutions, Figure S2: Liking vs. concentration functions for individual participants, vanilla milk, Figure S3: Descriptive information for individual optimal sucrose concentrations, Figure S4: Differences between experimental conditions in optimal sucrose concentration, Figure S5: Intensity vs. concentration functions for individual participants, aqueous solutions, Supplement Figure S6: Intensity vs. concentration functions for individual participants, vanilla milk, Table S1: Parameters for fits of rated liking vs. sucrose concentration to determine hedonically optimal (most liked) concentration.
