# Supplementary material for: Characterizing Individual Differences in Sweet Taste Hedonics: Test Methods, Locations, and Stimuli
Source: Nutrients. 2022 Jan 15;14(2):370. doi: 10.3390/nu14020370 (PMC8777740; doi:10.3390/nu14020370)
Supplement: Supplementary file 1 [file nutrients-14-00370-s001.zip › nutrients-1540906-supplementary.pdf]

nutrients MDPI

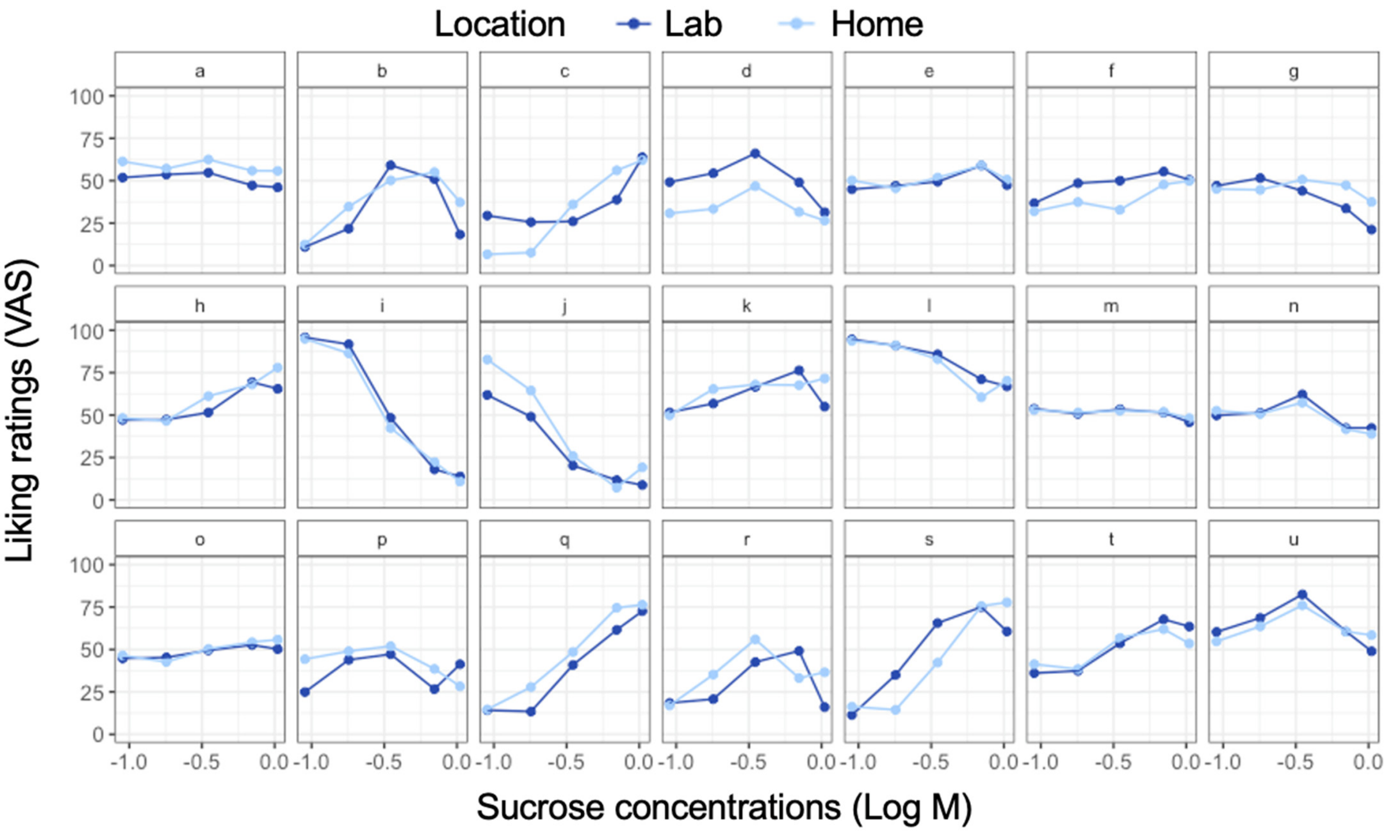

Figure S1. Liking vs. concentration functions for individual participants, aqueous solutions. Rated liking for aqueous solutions for individual participants, averaged across repeated test sessions. Letters at top of each panel are participant ID. X-axis: sucrose concentration, log M. Dark blue points and curves: measured in the laboratory. Light blue points and curves: measured at home.

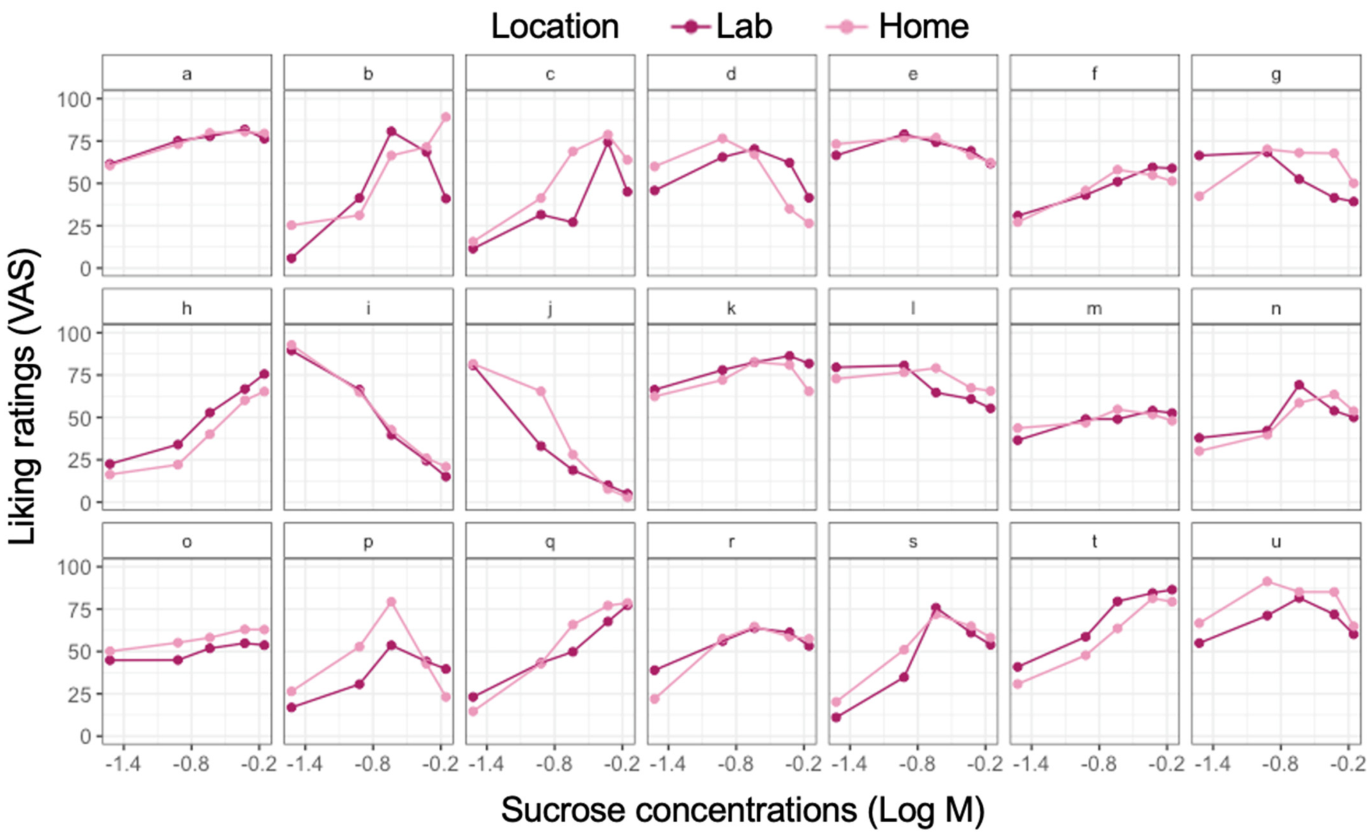

Figure S2. Liking vs. concentration functions for individual participants, vanilla milk. Rated liking for vanilla milk for individual participants, averaged across repeated test sessions. Letters at the top of each panel are participant ID. X-axis: sucrose concentration, log M. Red points and curves: measured in the laboratory. Pink points and curves: measured at home.

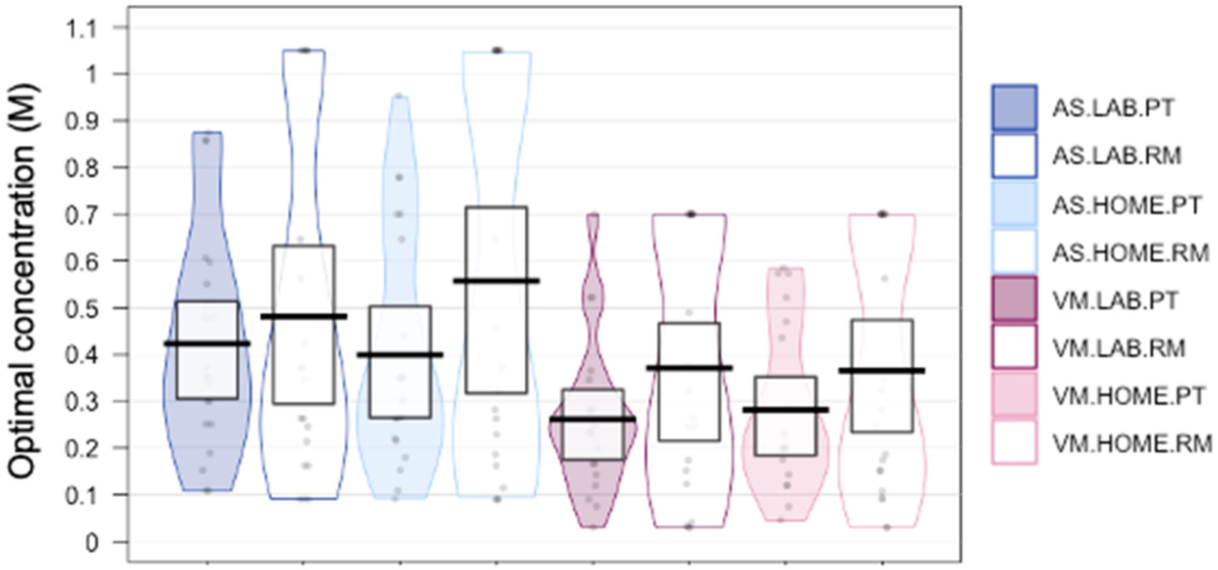

Figure S3. Descriptive information for individual optimal sucrose concentrations. Shape of the bean = data density; Center line within box = median; Upper and lower band around box = 95% confidence interval. Abbreviations: AS = aqueous solution; VM = vanilla milk; Lab = tests conducted in laboratory; Home = tests conducted at home; PT = preference tracking (optimal, or most preferred, concentration estimated via paired-comparison preference tracking); RM = rating method (optimal, or most liked, concentration estimated from ratings of liking).

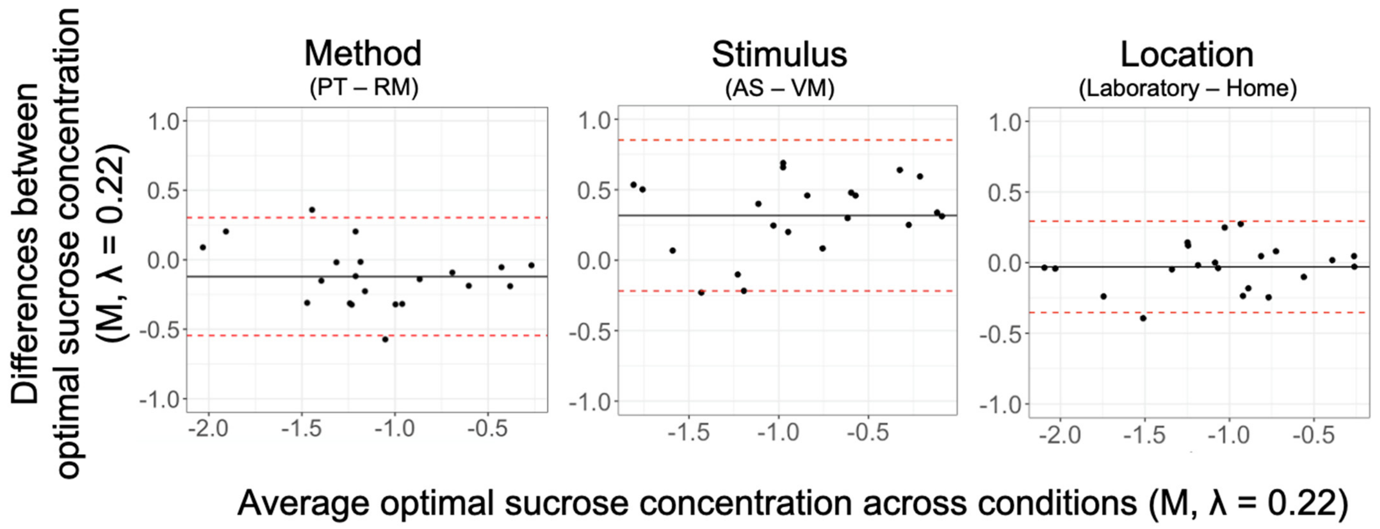

Figure S4. Differences between experimental conditions in optimal sucrose concentration (averaged across other conditions and replicate sessions) for individual participants. For example, points in the left-most panel represent optimal concentration estimated via paired-comparison preference tracking (PT) minus optimal concentration estimated from ratings of liking (RM), averaged across replicate sessions and other experimental conditions, for individual participants. Solid black line = mean difference (across individuals). Red dotted lines = 95% confidence interval. PT = Preference Tracking; RM = Rating Method; AS = Aqueous Solution; VM = Vanilla Milk. Units are differences in Box-Cox transformed (λ = 0.22) sucrose concentrations (M).

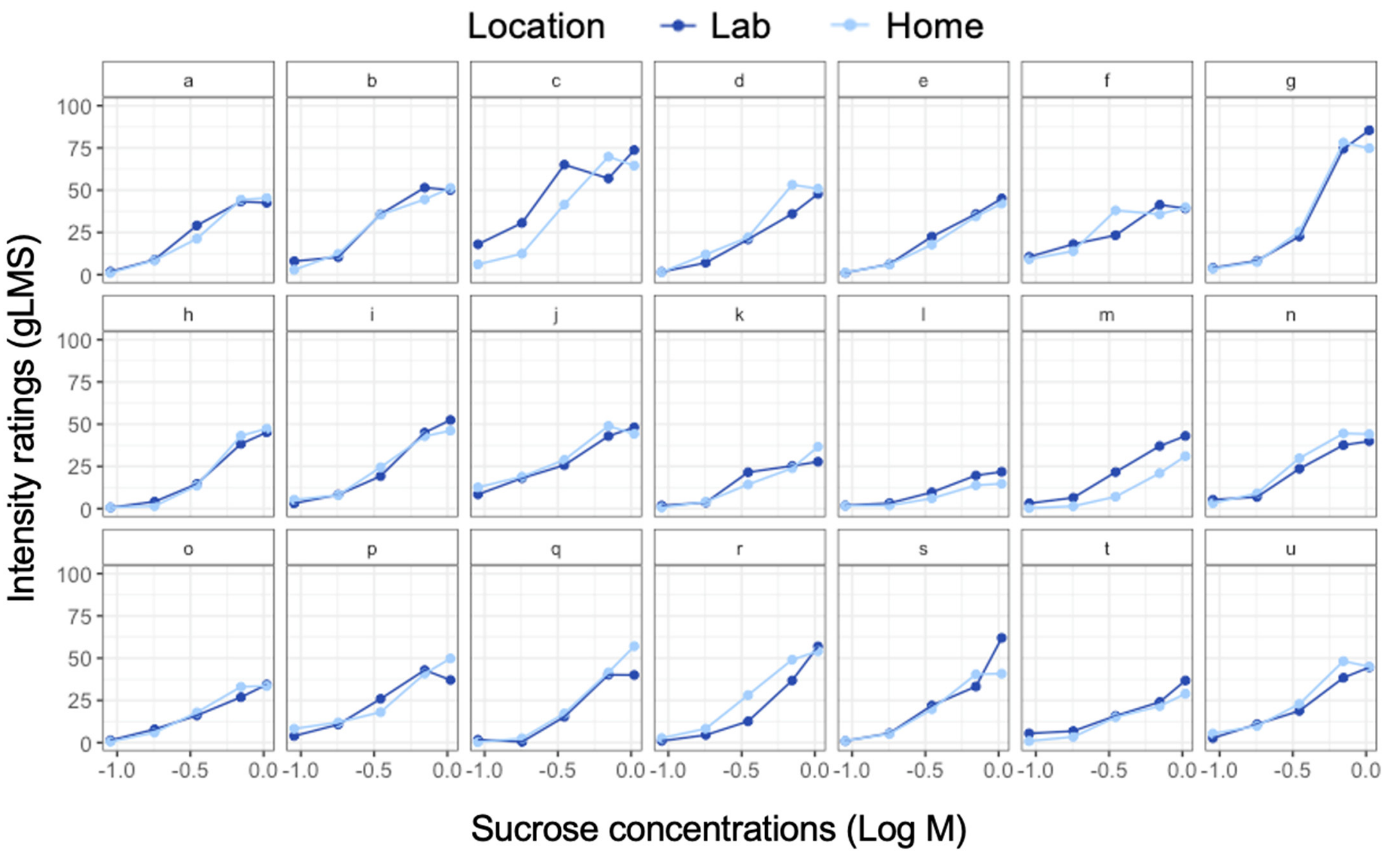

Figure S5. Intensity vs. concentration functions for individual participants, aqueous solutions. Rated intensity of aqueous solutions for individual participants, averaged across repeated test sessions. Letters at top of each panel are participant ID. X-axis: sucrose concentration, log M. Dark blue points and curves: measured in the laboratory. Light blue points and curves: measured at home.

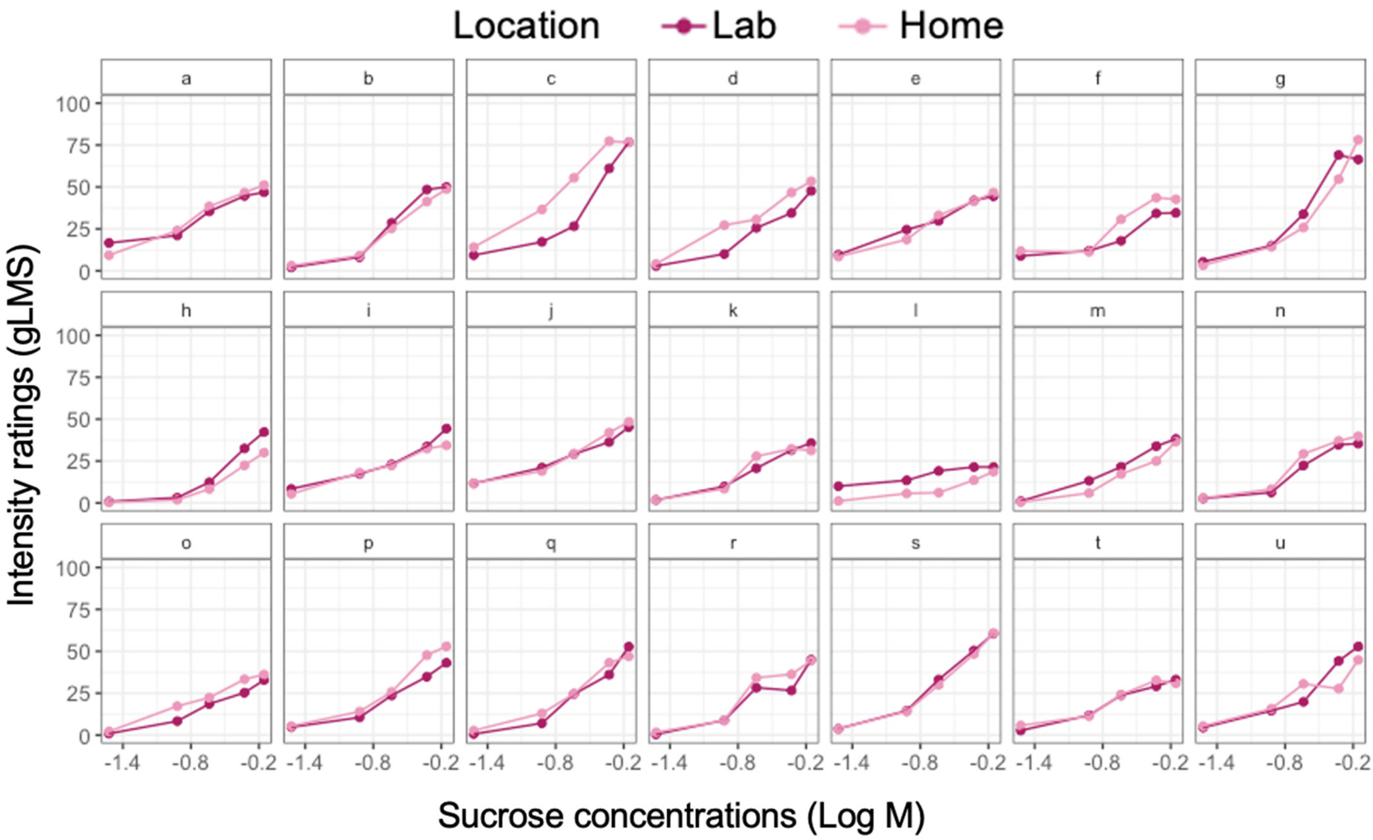

Figure S6. Intensity vs. concentration functions for individual participants, vanilla milk. Rated intensity of aqueous solutions for individual participants, averaged across repeated test sessions. Letters at top of each panel are participant ID. X-axis: sucrose concentration, log M. Red points and curves: measured in the laboratory. Pink points and curves: measured at home.

Table S1. Parameters for fits of rated liking vs. sucrose concentration to determine hedonically optimal (most liked) concentration.

Participant	Stimulus	Location	Session	Intercept	Linear	Quadratic	Cubic	r^2	Optimal
1	AS	HOME	1	59.72	0.00	0.00	0.00	0.00	0.18
1	AS	HOME	2	57.35	0.00	0.00	0.00	0.00	0.86
1	AS	LAB	1	44.63	-6.64	0.00	0.00	0.30	0.09
1	AS	LAB	2	59.20	0.00	-2.70	0.00	0.21	0.38
1	VM	HOME	1	82.71	8.52	-6.56	0.00	0.87	0.43
1	VM	HOME	2	75.23	4.06	-2.98	0.00	0.57	0.44
1	VM	LAB	1	83.74	0.00	-8.16	0.00	0.36	0.23
1	VM	LAB	2	75.37	7.57	-3.05	0.00	0.75	0.69
2	AS	HOME	1	51.94	-28.44	-20.73	18.74	0.67	0.25
2	AS	HOME	2	53.95	60.27	-12.09	-24.87	0.87	0.70
2	AS	LAB	1	62.67	48.38	-21.76	-24.22	0.82	0.61
2	AS	LAB	2	46.07	0.00	-25.30	0.00	0.72	0.38
2	VM	HOME	1	46.83	31.48	0.00	0.00	0.58	0.70
2	VM	HOME	2	65.32	33.54	0.00	-10.04	0.80	0.60
2	VM	LAB	1	71.76	60.04	-38.96	-32.92	0.92	0.37
2	VM	LAB	2	73.37	0.00	-20.14	8.84	0.71	0.23
3	AS	HOME	1	32.89	27.82	0.00	0.00	0.65	1.05
3	AS	HOME	2	36.54	56.23	0.00	-25.47	0.62	0.76
3	AS	LAB	1	17.73	0.00	23.04	0.00	0.42	1.05
3	AS	LAB	2	35.03	23.52	0.00	0.00	0.75	1.05
3	VM	HOME	1	71.53	0.00	-18.51	0.00	0.27	0.23
3	VM	HOME	2	63.72	56.59	-15.05	-18.48	0.88	0.48
3	VM	LAB	1	59.23	33.03	-27.73	-19.68	0.59	0.35
3	VM	LAB	2	29.26	49.96	9.07	-12.97	0.91	0.70
4	AS	HOME	1	22.57	0.00	0.00	0.00	0.00	0.49
4	AS	HOME	2	51.88	-8.77	-7.55	0.00	0.62	0.21
4	AS	LAB	1	54.08	-8.41	-12.15	0.00	0.82	0.27
4	AS	LAB	2	69.88	6.40	-13.82	-6.14	0.91	0.45
4	VM	HOME	1	67.92	-32.58	-10.78	9.71	0.82	0.09
4	VM	HOME	2	60.08	-36.81	-10.47	12.73	0.92	0.10
4	VM	LAB	1	71.43	0.00	-13.07	-6.58	0.65	0.23
4	VM	LAB	2	70.79	0.00	-19.09	0.00	0.55	0.23
5	AS	HOME	1	56.32	23.39	0.00	-12.34	0.71	0.72
5	AS	HOME	2	47.56	0.00	0.00	0.00	0.00	0.18
5	AS	LAB	1	63.39	14.42	-8.65	-3.61	0.94	0.63
5	AS	LAB	2	44.01	0.00	0.00	-3.00	0.29	0.09
5	VM	HOME	1	75.16	-4.93	-5.98	0.00	0.39	0.15
5	VM	HOME	2	75.89	-5.63	-3.61	0.00	0.83	0.09
5	VM	LAB	1	77.59	0.00	-5.68	0.00	0.27	0.23

5	VM	LAB	2	74.23	-9.69	-6.67	3.22	0.92	0.13
6	AS	HOME	1	38.88	0.00	8.83	0.00	0.30	1.05
6	AS	HOME	2	36.99	25.27	-3.47	-9.65	0.90	0.74
6	AS	LAB	1	50.16	0.00	0.00	0.00	0.00	0.35
6	AS	LAB	2	53.50	10.09	-7.98	0.00	0.74	0.64
6	VM	HOME	1	53.03	7.13	-7.38	0.00	0.79	0.37
6	VM	HOME	2	57.76	8.23	-10.28	0.00	0.68	0.35
6	VM	LAB	1	60.73	0.00	-6.56	3.91	0.80	0.23
6	VM	LAB	2	42.02	27.74	0.00	-8.18	0.93	0.60
7	AS	HOME	1	44.22	0.00	0.00	0.00	0.00	0.49
7	AS	HOME	2	45.87	0.00	0.00	0.00	0.00	0.25
7	AS	LAB	1	31.50	-13.95	0.00	0.00	0.80	0.09
7	AS	LAB	2	54.13	-6.57	-7.40	0.00	0.53	0.25
7	VM	HOME	1	66.37	0.00	-11.71	0.00	0.40	0.23
7	VM	HOME	2	78.54	0.00	-16.60	0.00	0.56	0.23
7	VM	LAB	1	36.54	-32.11	14.55	13.11	0.78	0.03
7	VM	LAB	2	72.70	-12.35	-14.99	0.00	0.85	0.15
8	AS	HOME	1	59.88	11.76	0.00	0.00	0.71	1.05
8	AS	HOME	2	58.03	12.27	3.25	0.00	0.90	1.05
8	AS	LAB	1	54.25	18.44	0.00	-7.76	0.83	0.78
8	AS	LAB	2	59.23	20.05	0.00	-6.60	0.91	0.84
8	VM	HOME	1	44.07	32.99	0.00	-8.05	0.89	0.65
8	VM	HOME	2	35.52	31.79	0.00	-7.56	0.96	0.66
8	VM	LAB	1	47.59	23.27	0.00	0.00	0.86	0.70
8	VM	LAB	2	53.05	18.06	0.00	0.00	0.86	0.70
9	AS	HOME	1	42.35	-55.69	5.56	13.19	0.97	0.09
9	AS	HOME	2	54.38	-32.87	0.00	0.00	0.71	0.09
9	AS	LAB	1	51.20	-61.50	0.00	17.48	0.88	0.12
9	AS	LAB	2	53.41	-53.39	0.00	13.16	0.92	0.10
9	VM	HOME	1	54.79	-25.07	0.00	0.00	0.88	0.03
9	VM	HOME	2	37.01	-28.95	7.86	0.00	0.97	0.03
9	VM	LAB	1	48.38	-29.94	0.00	0.00	0.87	0.03
9	VM	LAB	2	45.66	-27.15	0.00	0.00	0.74	0.03
10	AS	HOME	1	23.16	-26.38	20.91	0.00	0.82	0.09
10	AS	HOME	2	27.25	-47.81	10.69	11.77	0.94	0.09
10	AS	LAB	1	31.26	-16.20	0.00	0.00	0.42	0.09
10	AS	LAB	2	13.63	-27.83	17.51	0.00	0.98	0.09
10	VM	HOME	1	46.16	-63.14	0.00	18.07	0.97	0.06
10	VM	HOME	2	20.94	-36.47	11.32	5.59	0.98	0.03
10	VM	LAB	1	23.66	-26.61	10.72	0.00	0.93	0.03
10	VM	LAB	2	13.44	-7.83	12.30	-10.12	0.99	0.03
11	AS	HOME	1	65.88	0.00	0.00	6.22	0.76	1.05

11	AS	HOME	2	69.68	0.00	-8.14	3.72	0.52	0.38
11	AS	LAB	1	79.85	20.55	-19.32	-15.73	0.79	0.52
11	AS	LAB	2	61.68	17.78	0.00	-4.48	0.95	0.93
11	VM	HOME	1	82.93	3.98	-6.84	0.00	0.67	0.31
11	VM	HOME	2	81.05	21.20	-15.84	-14.77	0.55	0.35
11	VM	LAB	1	93.16	0.00	-6.03	0.00	0.41	0.23
11	VM	LAB	2	74.54	19.27	-5.71	-6.75	0.79	0.46
12	AS	HOME	1	75.91	-37.64	0.00	17.63	0.58	0.16
12	AS	HOME	2	82.06	-11.05	0.00	0.00	0.65	0.09
12	AS	LAB	1	88.32	-20.06	-2.95	5.53	0.96	0.14
12	AS	LAB	2	77.65	-10.26	0.00	0.00	0.95	0.09
12	VM	HOME	1	62.03	0.00	0.00	0.00	0.00	0.40
12	VM	HOME	2	86.68	-11.82	-4.54	0.00	0.76	0.04
12	VM	LAB	1	65.79	-9.01	0.00	0.00	0.63	0.03
12	VM	LAB	2	71.82	-25.31	0.00	9.19	0.79	0.07
13	AS	HOME	1	57.55	6.76	-3.34	-3.70	0.56	0.59
13	AS	HOME	2	48.60	-3.99	0.00	0.00	0.60	0.09
13	AS	LAB	1	56.55	0.00	-4.25	0.00	0.34	0.38
13	AS	LAB	2	49.53	0.00	0.00	-3.01	0.81	0.09
13	VM	HOME	1	48.46	0.00	0.00	0.00	0.00	0.33
13	VM	HOME	2	54.10	0.00	-5.02	0.00	0.55	0.23
13	VM	LAB	1	59.79	-10.75	-5.45	7.10	0.90	0.13
13	VM	LAB	2	42.50	10.44	0.00	0.00	0.64	0.70
14	AS	HOME	1	46.00	-6.91	0.00	0.00	0.76	0.09
14	AS	HOME	2	56.68	-3.89	-6.92	0.00	0.56	0.29
14	AS	LAB	1	56.71	-11.59	-7.82	6.26	0.58	0.24
14	AS	LAB	2	53.56	-3.95	-4.92	0.00	0.70	0.26
14	VM	HOME	1	54.28	28.28	-8.10	-10.08	0.83	0.47
14	VM	HOME	2	58.69	19.52	-10.77	-8.33	0.78	0.40
14	VM	LAB	1	62.57	0.00	-12.68	0.00	0.37	0.23
14	VM	LAB	2	55.44	0.00	-5.88	0.00	0.26	0.23
15	AS	HOME	1	52.35	13.09	0.00	-3.65	0.89	0.89
15	AS	HOME	2	47.79	0.00	0.00	0.00	0.00	0.31
15	AS	LAB	1	51.87	4.05	0.00	0.00	0.65	1.05
15	AS	LAB	2	45.13	0.00	0.00	0.00	0.00	0.49
15	VM	HOME	1	56.03	6.42	0.00	0.00	0.67	0.70
15	VM	HOME	2	59.71	3.71	0.00	0.00	0.26	0.70
15	VM	LAB	1	52.64	15.27	0.00	-5.76	0.64	0.55
15	VM	LAB	2	46.73	0.00	0.00	0.00	0.00	0.40
16	AS	HOME	1	45.28	22.58	-12.22	-10.21	0.90	0.60
16	AS	HOME	2	51.37	-20.32	0.00	0.00	0.65	0.09
16	AS	LAB	1	35.18	0.00	0.00	0.00	0.00	0.61

16	AS	LAB	2	38.44	0.00	0.00	0.00	0.00	0.25
16	VM	HOME	1	60.50	-11.80	-15.60	0.00	0.51	0.15
16	VM	HOME	2	74.07	0.00	-34.14	0.00	0.84	0.23
16	VM	LAB	1	57.95	0.00	-17.15	0.00	0.37	0.23
16	VM	LAB	2	36.58	29.73	-7.38	-12.44	0.62	0.46
17	AS	HOME	1	62.17	34.65	-11.18	-7.84	0.96	0.74
17	AS	HOME	2	45.32	28.47	0.00	0.00	0.96	1.05
17	AS	LAB	1	45.53	23.89	0.00	0.00	0.79	1.05
17	AS	LAB	2	29.68	34.66	7.13	-6.68	0.99	1.05
17	VM	HOME	1	57.15	22.32	-6.93	0.00	0.85	0.70
17	VM	HOME	2	69.16	24.46	-9.40	0.00	0.78	0.70
17	VM	LAB	1	53.07	16.07	0.00	0.00	0.54	0.70
17	VM	LAB	2	51.40	23.77	0.00	0.00	0.87	0.70
18	AS	HOME	1	63.25	-21.01	-20.35	17.07	0.84	0.27
18	AS	HOME	2	32.16	8.84	-8.09	0.00	0.61	0.60
18	AS	LAB	1	51.96	31.97	-24.57	-18.84	0.80	0.55
18	AS	LAB	2	31.64	25.21	0.00	-14.98	0.35	0.70
18	VM	HOME	1	60.95	-24.38	-6.75	20.24	0.89	0.13
18	VM	HOME	2	66.03	24.83	-16.87	-8.09	0.96	0.39
18	VM	LAB	1	65.88	28.41	-12.57	-9.24	0.82	0.44
18	VM	LAB	2	53.68	0.00	0.00	0.00	0.00	0.17
19	AS	HOME	1	45.74	49.97	0.00	-11.52	0.94	0.96
19	AS	HOME	2	47.21	48.41	0.00	-18.45	0.87	0.80
19	AS	LAB	1	85.23	14.88	-34.26	0.00	0.94	0.46
19	AS	LAB	2	44.68	28.78	0.00	0.00	0.86	1.05
19	VM	HOME	1	46.48	0.00	0.00	0.00	0.00	0.33
19	VM	HOME	2	76.06	18.21	-17.81	0.00	0.89	0.38
19	VM	LAB	1	60.83	0.00	-20.68	0.00	0.34	0.23
19	VM	LAB	2	68.36	45.58	-20.08	-17.20	0.77	0.42
20	AS	HOME	1	48.48	24.60	0.00	-15.15	0.37	0.69
20	AS	HOME	2	59.12	11.89	-6.20	0.00	0.74	0.81
20	AS	LAB	1	48.83	41.24	0.00	-14.95	0.98	0.81
20	AS	LAB	2	60.51	6.23	-5.35	0.00	0.48	0.62
20	VM	HOME	1	64.43	28.63	0.00	-8.32	0.92	0.61
20	VM	HOME	2	55.71	23.90	0.00	0.00	0.66	0.70
20	VM	LAB	1	74.13	39.30	-7.29	-11.82	0.89	0.52
20	VM	LAB	2	77.69	5.88	-6.86	4.21	0.97	0.70
21	AS	HOME	1	70.73	0.00	-3.74	4.01	0.78	1.05
21	AS	HOME	2	71.51	0.00	-15.37	0.00	0.45	0.38
21	AS	LAB	1	76.70	0.00	-10.81	0.00	0.32	0.38
21	AS	LAB	2	75.34	-11.69	-15.49	0.00	0.69	0.27
21	VM	HOME	1	93.62	0.00	-10.01	0.00	0.59	0.23

21	VM	HOME	2	86.65	0.00	-15.55	0.00	0.32	0.23
21	VM	LAB	1	77.46	17.91	-13.02	-11.28	0.44	0.36
21	VM	LAB	2	81.34	0.00	-13.98	0.00	0.35	0.23

[1] Model beverage. AS = aqueous solutions; VM = vanilla milk. [2] Test location. HOME = test conducted at home; LAB = test conducted in the laboratory. [3] Replicate sessions (first and second). [4] Model fit parameter, intercept. [5] Model fit parameter, linear term. A value of 0 indicates that the best fit model did not include a linear term. [6] Model fit parameter, quadratic term. A value of 0 indicates that the best fit model did not include a quadratic term. [7] Model fit parameter, cubic term. A value of 0 indicates that the best fit model did not include a cubic term. [8] Proportion of variance for which the model fit accounts. A value of 0 indicates an intercept-only model. [9] Most liked concentration of sucrose (M). Local maximum of fitted function within range of presented concentrations. For intercept-only fits, this value is the geometric mean of the two highest rated concentrations.
